# Polymorphisms in folate pathway and pemetrexed treatment outcome in patients with malignant pleural mesothelioma

**DOI:** 10.2478/raon-2013-0086

**Published:** 2014-04-25

**Authors:** Katja Goricar, Viljem Kovac, Vita Dolzan

**Affiliations:** 1 Pharmacogenetics Laboratory, Institute of Biochemistry, Faculty of Medicine, University of Ljubljana, Ljubljana, Slovenia; 2 Institute of Oncology Ljubljana, Ljubljana, Slovenia

**Keywords:** mesothelioma, pemetrexed, polymorphism, folate pathway, toxicity

## Abstract

**Introduction:**

A combination of pemetrexed and cisplatin has been shown to improve the outcome in patients with malignant pleural mesothelioma (MPM), however, there is a great heterogeneity in treatment response among patients. The aim of our study was to evaluate the influence of polymorphisms in folate pathway and transporter genes on pemetrexed treatment outcome in Slovenian patients with MPM.

**Methods:**

MPM patients treated with pemetrexed in the course of a prospective randomized clinical trial were genotyped for nineteen polymorphisms in five genes of folate pathway and six transporter genes. Logistic regression was used to assess the influence of polymorphisms on treatment efficacy and toxicity, while Cox regression was used to determine their influence on progression-free and overall survival.

**Results:**

Patients with at least one polymorphic *MTHFD1* rs2236225 allele had a significantly lower response rate (*p* = 0.005; odds ratio [OR] = 0.12; 95% confidence interval [CI] = 0.03−0.54) and shorter progression-free survival (*p* = 0.032; hazard ratio [HR] = 3.10; 95% CI = 1.10−8.74) than non-carriers. Polymorphisms in transporter genes did not influence survival; however, several were associated with toxicity. Liver toxicity was significantly lower in carriers of polymorphic *ABCC2* rs2273697 (*p* = 0.028; OR = 0.23; 95% CI = 0.06−0.85), *SLCO1B1* rs4149056 (*p* = 0.028; OR = 0.23; 95% CI = 0.06−0.85) and rs11045879 (*p* = 0.014; OR = 0.18; 95% CI = 0.05−0.71) alleles compared to non-carriers, as well as in patients with *SLCO1B1* GCAC haplotype (*p* = 0.048; OR = 0.17; 95% CI = 0.03−0.98). Gastrointestinal toxicity was much more common in patients with polymorphic *ABCC2* rs717620 allele (*p* = 0.004; OR = 10.67; 95% CI = 2.15−52.85) and *ABCC2* CAG haplotype (*p* = 0.006; OR = 5.67; 95% CI = 1.64−19.66).

**Conclusions:**

*MTHFD1* polymorphism affected treatment response and survival, while polymorphisms in *ABCC2* and *SLCO1B1* transporter genes influenced the risk for toxicity. These polymorphisms could serve as potential markers of pemetrexed treatment outcome in patients with MPM.

## Introduction

Malignant pleural mesothelioma (MPM) is a rare but aggressive tumour of mesothelial surfaces of pleura that is mainly connected with asbestos exposure.^1^ Most patients are diagnosed in later stages, often with unresectable disease, therefore the prognosis is poor and median survival time is rarely above two years.^2^,^3^ Several clinical and genetic factors can have a prognostic value in MPM, but successful treatment remains challenging.^2^ Most patients with MPM are treated with systemic chemotherapy; usually either pemetrexed (PMX) or gemcitabine combined with a platinum agent, which are often used in different oncological diseases.^4^–^6^ Studies have shown that chemotherapy significantly improves survival of patients with MPM. Randomized clinical trial has shown that treatment with combination of PMX and cisplatin improves outcome in patients with MPM^7^–^9^ and comparable results were obtained for treatment with gemcitabine and cisplatin.^3^,^4^,^10^

PMX is a folic acid analogue, frequently used in treatment of MPM or non-small cell lung cancer (NSCLC). It inhibits several key folate pathway enzymes, including thymidylate synthase (TYMS), thus leading to impaired DNA synthesis.^11^ Several other enzymes such as *MTHFD1*, *MTHFR*, *MTR*, and *MTRR*, participate in folate metabolic pathway and may influence treatment with antifolates.^12^

The efficacy of PMX may also depend on its transport into and out of the cell. The most important for PMX uptake is reduced folate carrier (SLC19A1), but hepatic SLCO1B1 transporter is also involved in antifolate transport.^13^ On the other hand, ATP-binding cassette (ABC) transporters are involved in efflux of PMX from the cells.^14^,^15^

Response rate to PMX treatment is up to 40% in patients with MPM^3^ and toxicity of PMX may be dose limiting. Differences in response to treatment with PMX may be partly due to genetic variability of enzymes involved in its transport and metabolism. Single nucleotide polymorphisms (SNPs) in folate pathway and folate transporter genes have been extensively studied regarding their impact on toxicity and efficacy of methotrexate, the most common antifolate used in chemotherapy. Regarding PMX, a newer antifolate agent, only a few studies of *TYMS*, *MTHFR*, and *SLC19A1* polymorphisms in NSCLC were published to date.^16^,^17^ Very little is known about the influence of these polymorphisms on response to treatment with PMX in MPM.^18^

The aim of this study was therefore to evaluate how polymorphisms in folate pathway genes (*TYMS*, *MTHFR*, *MTHFD1*, *MTR*, *MTRR*) and transporter genes (*SLC19A1*, *SLCO1B1*, *ABCB1*, *ABCC2*, *ABCG2*) affect treatment outcome, toxicity and survival in Slovenian patients with MPM, treated with PMX.

## Patients and methods

### Patients

Patients eligible for inclusion in the pharmacogenetic study had histologically proven MPM and were participating in an ongoing prospective randomised phase II clinical trial “Cisplatin with either Alimta or Gemcitabine in long infusion (AGILI) for mesothelioma” (Trial registration ID: NCT01281800) at the Institute of Oncology Ljubljana, Slovenia.^19^ Patients that meet the trial’s inclusion criteria were randomized in two groups, receiving either PMX or gemcitabine in combination with cisplatin.^19^ Patients in which cisplatin was substituted with carboplatin due to poor performance status or renal dysfunction were also included. If disease progression occurred, chemotherapy regimen was switched in second line.

Patients were diagnosed mostly at the University Clinic of Pulmonary and Allergic Diseases in Golnik, Slovenia and treated at the Institute of Oncology Ljubljana, Slovenia. Clinical data were obtained from the medical records or assessed during the clinical interview.

The study was approved by the Slovenian Ethics Committee for Research in Medicine and was carried out according to the Declaration of Helsinki.

### Response, survival and toxicity assessment

Tumour response was evaluated using modified Response Evaluation Criteria in Solid Tumours (RECIST).^20^ Response rate was defined as the percentage of patients achieving partial or complete response. Progression-free survival (PFS) and overall survival (OS) were evaluated in survival analysis.

Haematological toxicity (anaemia, leucopoenia, neutropenia, and thrombocytopenia), liver toxicity, renal toxicity, and gastrointestinal (GI) toxicity were evaluated according to National Cancer Institute Common Terminology Criteria for Adverse Events, version 4.0.^21^

### DNA extraction and genotyping

Peripheral blood samples were collected before the first day of the treatment. Extraction of genomic DNA from frozen whole-blood samples was performed according to the manufacturer’s instructions using Qiagen FlexiGene kit (Qiagen, Hilden, Germany).

We genotyped 19 different polymorphisms in five folate pathway and six transporter genes. *MTHFD1* rs2236225 (Arg653Gln), *MTHFR* rs1801133 (Ala222Val) and rs1801131 (Glu429Ala), *SLCO1B1* rs2306283 (Asn130Asp), and *ABCB1* rs1045642 (Ile1145Ile) polymorphisms were determined using TaqMan SNP Genotyping assays according to the manufacturer’s instructions (Applied Biosystems, Foster City, CA) as previously described.^22^ Genotyping of *MTRR* rs1801394 (Ile22Met), *MTR* rs1805087 (Asp919Gly), *SLC19A1* rs1051266 (Arg27Cys), *SLCO1B1* rs11045879 (intronic), rs4149056 (Val174Ala) and rs2900478 (intronic), *ABCC2* rs717620 (5’ untranslated region (UTR) -24C>T), rs2273697 (Val417Ile) and rs2804402 (5’ UTR -1019A>G), *ABCC4* rs2274407 (Lys304Asn), and *ABCG2* rs2231142 (Gln141Lys) and rs2231137 (Val12Met) polymorphisms was carried out using a fluorescence-based competitive allele-specific (KASPar) assay according to the manufacturer’s instructions (KBiosciences, Herts, UK). Determination of promoter *TYMS* rs34743033 (5’ UTR 2R>3R) polymorphism^23^ and *ABCB1* rs2032582 (Ala893Ser/Thr) polymorphism was carried out using PCR amplification followed by the analysis of PCR fragments on agarose gel as previously described.^24^

### Statistical analyses

Median and range (minimum-maximum) were used to present central tendency and variability. To assess deviation from Hardy–Weinberg equilibrium (HWE), standard chi-square test was used. A dominant genetic model was used in all statistical analyses. The influences of genetic polymorphisms on treatment outcome were examined by univariable logistic regression to calculate odds ratios (ORs) and their 95% confidence intervals (CIs). In survival analysis Cox proportional hazards model was used and the hazard ratio (HR) with the 95% CI was determined. All potential clinical and treatment predictors were also independently analysed for their influence on treatment outcome. All statistical analyses were carried out by Statistical Package for the Social Sciences (SPSS) for Windows, version 19.0 (IBM Corporation, Armonk, NY, USA).

Haplotypes were reconstructed and analysed using Thesias software^25^ as described previously.^26^ Only haplotypes with frequencies above 5% were included in the statistical analyses and the most frequent haplotype was used as reference.

All statistical tests were two-sided and the level of significance was set to 0.05. Due to the exploratory nature of the study, no adjustments for multiple comparisons were used.

## Results

### Patients’ characteristics

In our study, we included 41 patients with MPM, participating in the AGILI trial from 2008 to December 2012. In total, 29 patients received PMX as first line of chemotherapy and 12 received PMX as second line. Clinical characteristics of the study group are summarized in Table 1. Twenty (48.8%) patients were smokers and 33 (80.5%) were either occupationally or environmentally exposed to asbestos. Median C-reactive protein (CRP) level at diagnosis was 23 mg/l (range: 1−192 mg/l). The majority of patients (70.7%) received at least 4 cycles of chemotherapy. To the date of the analysis, disease progression occurred in 32 (78.0%) patients and 23 patients (56.1%) had died. There were no significant differences between patients receiving PMX as the first or second line of chemotherapy (Table 1).

### Genotyping analysis

All genotype frequencies (Supplemental Table 1) were in agreement with HWE (*p* > 0.05). Three SNPs were excluded from further statistical analyses: *SLCO1B1* rs2900478 was in complete linkage disequilibrium (LD) with *SLCO1B1* rs11045879, while *ABCG2* rs2231137 and *ABCC4* rs2274407 were too rare.

### Tumour response analysis

Among 41 patients, one (2.4%) was a complete responder and 12 (29.3%) were partial responders, meaning the overall response rate was 31.7%. Nineteen patients had stable disease and nine had progressive disease. Data on the influence of polymorphisms on response rate are presented in Table 2. Patients with at least one polymorphic *MTHFD1* rs2236225 allele had lower response rate (*p* = 0.005; OR = 0.12; 95% CI = 0.03−0.54) than patients with two wild-type alleles. On the other hand, patients with at least one polymorphic *ABCC2* rs2273697 had significantly better response rate than non-carriers (*p* = 0.031; OR = 4.75; 95% CI = 1.15−19.65).

Higher TNM stage was the only clinical parameter significantly associated with lower response rate (*p* = 0.013; OR = 0.35; 95% CI = 0.15−0.79). After adjustment for TNM stage, *MTHFD1* rs2236225 remained associated with response rate (*p* = 0.016; OR = 0.14; 95% CI = 0.03−0.70), but the effect of *ABCC2* rs2273697 was no longer significant (*p* = 0.065; OR = 4.32; 95% CI = 0.91−20.43).

### Toxicity analysis

Several polymorphisms in transporter genes influenced occurrence of treatment-related toxicities. *ABCC2* rs2273697 conferred protection against development of any toxicity (*p* = 0.035; OR = 0.09; 95% CI = 0.01−0.85). Liver toxicity was significantly less frequent in carriers of polymorphic *SLCO1B1* rs11045879 (*p* = 0.014; OR = 0.18; 95% CI = 0.05−0.71) and rs4149056 (*p* = 0.028; OR = 0.23; 95% CI = 0.06−0.85) alleles. *ABCC2* rs2273697 was also associated with decreased liver toxicity (*p* = 0.028; OR = 0.23; 95% CI = 0.06−0.85). On the other hand, GI toxicity was much more common in patients with polymorphic *ABCC2* rs717620 allele (*p* = 0.004; OR = 10.67; 95% CI = 2.15–52.85). The other investigated polymorphisms in transporter and folate pathway genes did not significantly affect occurrence of either overall or specific toxicity (Table 3 and Supplemental Table 2).

### Survival analysis

Analysis of OS was performed in the whole cohort of patients, while PFS was only analysed in the group of patients receiving PMX in the first line treatment. Firstly, clinical and treatment characteristics were examined for their influence on PFS or OS. Increased level of CRP before the first day of the treatment was the only parameter associated both with shorter PFS and shorter OS (*p* = 0.002, HR = 1.015, 95% CI = 1.01−1.03 and *p* < 0.001, HR = 1.015, 95% CI = 1.01−1.02, respectively). Patients with sarcomatoid or biphasic MPM had significantly shorter PFS (*p* = 0.026, HR = 4.59, 95% CI = 1.20−17.52). On the other hand, if patients received more chemotherapy cycles, OS was longer (*p* = 0.024, HR = 0.36, 95% CI = 0.15−0.88). However, only CRP remained significant in multivariable model.

**FIGURE 1.**
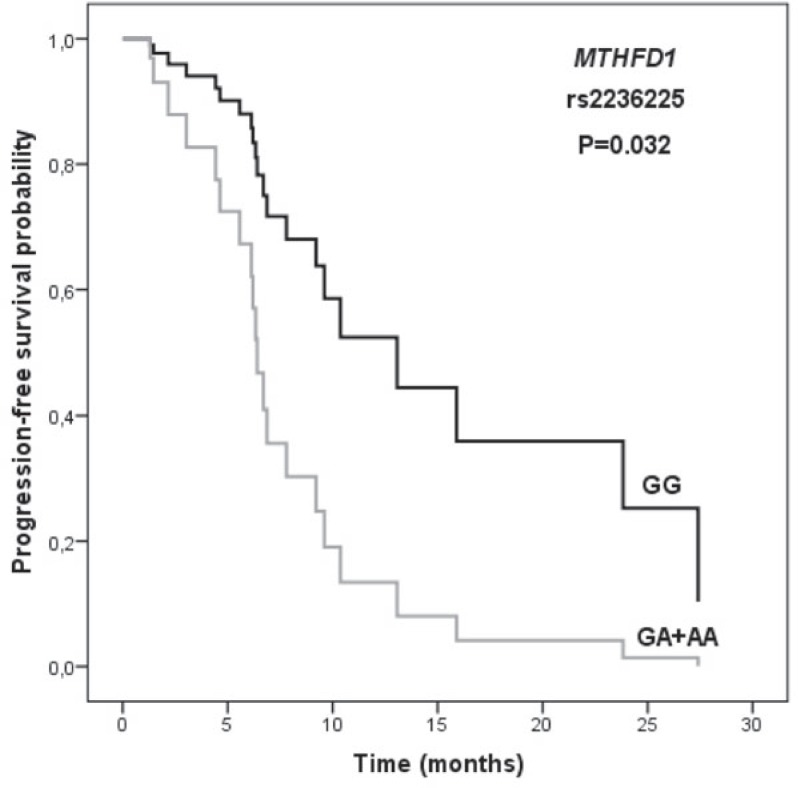
The influence of *MTHFD1* rs2236225 polymorphism on progression-free survival in patients with malignant pleural mesothelioma. *P*-value was calculated using Cox regression and adjusted for C-reactive protein level.

The data on the influence of SNPs on survival is presented in Table 2. Among the investigated polymorphisms, only *MTHFD1* rs2236225 significantly influenced PFS (*p* = 0.032; HR = 3.10; 95% CI = 1.10−8.74) after adjustment for CRP level (Figure 1). MPM patients with at least one polymorphic *MTHFD1* allele and high CRP level had shorter survival than patients with two wild-type alleles and lower CRP level.

### Haplotype analysis

Haplotype analysis was performed to assess the combined effect of SNPs within one gene. Three *SLCO1B1* haplotypes (ATT, GCC, and GTT) had frequencies above 5% and covered approximately 98% of variability within this gene (Table 4). Liver toxicity was less common in patients with GCC haplotype. This haplotype included all the polymorphic alleles, associated with decreased liver toxicity in single SNP analysis, compared with the reference ATT haplotype (*p* = 0.048; OR = 0.17; 95% CI = 0.03−0.98).

Four *ABCC2* haplotypes (CGG, CAG, TGG and TGA) covered all the variability within this gene (Table 4). GI toxicity was significantly more common in patients with CAG haplotype (*p* = 0.006; OR = 5.67; 95% CI = 1.64−19.66) with polymorphic rs717620 allele, associated with increased GI toxicity in single SNP analysis.

## Discussion

Patients with MPM participating in a prospective randomized clinical trial were investigated for the influence of folate pathway and transporter polymorphisms on PMX treatment response. Among folate pathway genes only *MTHFD1* was associated with response rate and survival, while transporters mainly influenced PMX-related toxicity.

Only few pharmacogenetic studies on PMX treatment have been published so far. The only study in MPM focused on *TYMS* rs34743033, a tandem repeat of 28 base pairs in the 5′ UTR promoter region that changes TYMS mRNA and protein expression.^18^ Consistent with our results, no association of this polymorphism with treatment outcome was observed, even though both mRNA and protein expression significantly affected survival.^18^,^27^,^28^ Similar results were obtained in NSCLC.^17^,^29^–^32^

In our study, the carriers of the polymorphic *MTHFD1* rs2236225 allele had a significantly shorter PFS and were less likely to achieve complete or partial response, even after adjustment for TNM stage. MTHFD1 is essential for the generation of methylene-THF required for thymidylate synthesis and *MTHFD1* rs2236225 (Arg653Gln) was shown to reduce the enzyme activity^33^, leading to increased levels of methylene-THF and reducing cytotoxic effects of PMX.^23^,^34^ To our knowledge, no previous studies investigated the role of *MTHFD1* SNPs in treatment with PMX. However, in some, but not all studies on methotrexate treatment in acute lymphoblastic leukaemia (ALL), the variant allele was associated with shorter event-free survival.^23^,^34^

In our MPM patients PMX treatment outcome was not influenced by other investigated folate pathway SNPs. Most of them have not been studied yet in PMX treated MPM patients, however in NSCLC polymorphic *MTHFR* rs1801133 allele conferred to increased survival in a recessive model.^17^,^31^

To our knowledge, this is the first study investigating folate transporter gene polymorphisms regarding treatment outcome in MPM. Patients with at least one polymorphic *ABCC2* rs2273697 allele had better response rate and less overall and liver toxicity than non-carriers, while polymorphic *ABCC2* rs717620 allele and *ABCC2* CAG haplotype conferred to increased GI toxicity. *ABCC2* encodes one of the multidrug resistance associated transporters, involved in transport of both natural folates and antifolate agents.^35^ The knowledge about the functional significance of *ABCC2* SNPs is limited, but rs717620 was associated with decreased promoter activity and mRNA expression of *ABCC2*, possibly affecting the accumulation of PMX in cells.^15^,^36^ However, *ABCC2* is also involved in transport of platinum compounds and this could contribute to its effect on treatment response. Indeed, some studies have shown that rs717620 affects treatment with platinum agents in lung cancer.^37^,^38^

In the present study, polymorphic *SLCO1B1* rs11045879 and rs4149056 alleles were significantly associated with liver toxicity both in single SNP and haplotype analysis. *SLCO1B1* encodes one of the main influx transporters expressed on the basolateral membrane of hepatocytes involved in uptake and clearance of many endogenous compounds and drugs, such as methotrexate.^39^ *SLCO1B1* rs4149056 and rs2306283 were associated with decreased membrane expression and activity of the transporter.^39^,^40^ Rs11045879 was identified in a genome-wide association study as the most important genetic factor associated with lower methotrexate clearance in patients with ALL. In later studies, a similar association with methotrexate clearance was observed for rs4149056.^40^,^41^

Although *SLC19A1* is the predominant uptake transporter for antifolates, we observed no impact of rs1051266 on PMX treatment. Our results are consistent with studies on NSCLC^17^,^31^, although one study reported several other *SLC19A1* SNPs associated with OS.^16^

Our study represents the first comprehensive pharmacogenetic study of treatment with PMX in MPM patients participating in a prospective randomized trial. As MPM is a rare cancer, some potential limitations of our study arise from its small sample size, such as low statistical power. However, the strength of our study was that all patients were from a homogenous population^42^, included in phase II clinical trial with well-defined inclusion criteria and clinical protocol, and treated in the same facility, thus minimizing the impact of other variables. We also evaluated the influence of potentially important clinical parameters and included haplotype analysis to evaluate the combined influence of more SNPs in one gene.

Both PMX and gemcitabine have shown comparable efficacy in MPM treatment.^3^,^4^ In previous studies, we have identified some polymorphisms that influence MPM treatment with gemcitabine and cisplatin.^26^,^43^,^44^ Our present results show for the first time that *MTHFD1*, *ABCC2*, and *SLCO1B1* polymorphisms may play an important role in PMX treatment response in patients with MPM. These SNPs could serve as biomarkers for more personalized treatment and in the future, selection of treatment based on genetic factors may contribute to better treatment outcomes in patients with MPM.

## Supplementary files

**Supplemental Table 1**. Distribution of genotype frequencies in patients with malignant pleural mesothelioma (N = 41). Available from: http://www.degruyter.com/view/j/raon.2014.48.issue-2/raon-2013-0086/suppl/raon-2013-0086_supp1.pdf

**Supplemental Table 2** The influence of selected polymorphisms on overall toxicity, hematological and renal toxicity. Available from: http://www.degruyter.com/view/j/raon.2014.48.issue-2/raon-2013-0086/suppl/raon-2013-0086_supp2.pdf

## Figures and Tables

**TABLE 1.** Clinical and treatment characteristics of patients with malignant pleural mesothelioma receiving pemetrexed (PMX) chemotherapy

**Characteristic**		**All patients (N = 41)**	**PMX first line (N = 29)**	**PMX second line (N = 12)**	**p**

		**N (%)**	**N (%)**	**N (%)**	
**Gender**	Male	33 (80.5)	22 (75.9)	11 (91.7)	0.398^a^
	Female	8 (19.5)	7 (24.1)	1 (8.3)	
**Stage**	I	3 (7.3)	3 (10.3)	0	0.578^a^
	II	12 (29.3)	9 (31.0)	3 (25.0)	
	III	12 (29.3)	9 (31.0)	3 (25.0)	
	IV	14 (34.1)	8 (27.6)	6 (50.0)	
**Histological type**	Epitheloid	33 (80.5)	25 (86.2)	8 (66.7)	0.267^a^
	Biphasic	3 (7.3)	1 (3.4)	2 (16.7)	
	Sarcomatoid	3 (7.3)	2 (6.9)	1 (8.3)	
	Not characterized	2 (4.9)	1 (3.4)	1 (8.3)	
**ECOG performance status**	0	20 (48.8)	15 (51.7)	5 (41.7)	0.499^a^
	1	18 (43.9)	11 (37.9)	7 (58.3)	
	2	3 (7.3)	3 (10.3)	0	
**Response rate**	CR and PR	13 (31.7)	11 (37.9)	2 (16.7)	0.140^a^
	SD	19 (46.3)	14 (48.3)	5 (41.7)	
	Progress	9 (22.0)	4 (13.8)	5 (41.7)	
**Liver toxicity**	Present	24 (58.5)	15 (51.7)	9 (75.0)	0.296^a^
	Absent	17 (41.5)	14 (48.3)	3 (25.0)	
**GI toxicity**	Present	11 (26.8)	9 (31.0)	2 (16.7)	0.457^a^
	Absent	30 (73.2)	20 (69.0)	10 (83.3)	
**Haematological toxicity**	Present	19 (46.3)	13 (44.8)	6 (50.0)	1.000^a^
	Absent	22 (53.7)	16 (55.2)	6 (50.0)	
**Renal toxicity**	Present	18 (43.9)	13 (44.8)	5 (41.7)	1.000^a^
	Absent	23 (56.1)	16 (55.2)	7(58.3)	
**Overall toxicity**	Present	34 (82.9)	23 (79.3)	11 (91.7)	0.419^a^
	Absent	7 (17.1)	6 (20.7)	1 (8.3)	
**Overall survival**	median (min-max)	12.1 (1.0–36.1)	11.9 (1.0–36.1)	14.4 (6.4–31.6)	0.558^b^
**Progression-free survival**	median (min-max)	6.5 (1.0–36.1)	6.7 (1.0–36.1)	6.4 (6.4–31.6)	0.431^b^
**Age**	median (min-max)	63 (35–79)	63 (35–79)	64 (46–78)	0.785^b^

a calculated using Fisher’s exact test;

b calculated using Mann-Whitney U-test; CR = complete response; PR = partial response; SD = stable disease; ECOG = Eastern Cooperative Oncology Group; GI = gastrointestinal

**TABLE 2.** The influence of investigated polymorphisms on response rate, overall survival (N = 41) and progression-free survival (N = 29) in patients with malignant pleural mesothelioma

**Gene**	**Polymorphism**		**Response rate^a^**		**Progression-free survival^b^**		**Overall survival^b^**	

			**OR (95 % CI)**	**p**	**HR (95 % CI)**	**p**	**HR (95 % CI)**	**p**
***MTHFR***	rs1801133	CC	Reference		Reference		Reference	
		CT+TT	0.63 (0.16–2.39)	0.492	1.82 (0.69–5.04)	0.248	1.11 (0.47–2.64)	0.809
	rs1801131	AA	Reference		Reference		Reference	
		AC+CC	0.76 (0.20–2.85)	0.678	0.93 (0.37–2.34)	0.880	1.15 (0.49–2.74)	0.746
***MTHFD1***	rs2236225	GG	Reference		Reference		Reference	
		GA+AA	0.12 (0.03–0.54)	**0.005**	3.10 (1.10–8.74)	**0.032**	1.81 (0.72–4.57)	0.207
***TYMS***	rs34743033	2R/2R	Reference		Reference		Reference	
		2R/3R+3R/3R	0.47 (0.12–1.83)	0.274	0.77 (0.28–2.08)	0.605	0.62 (0.23–1.66)	0.336
***MTRR***	rs1801394	AA	Reference		Reference		Reference	
		AG+GG	0.91 (0.19–4.39)	0.906	1.14 (0.31–4.20)	0.841	0.57 (0.18–1.78)	0.334
***MTR***	rs1805087	AA	Reference		Reference		Reference	
		AG+GG	0.97 (0.25–3.73)	0.960	1.38 (0.57–3.32)	0.473	1.57 (0.66–3.72)	0.310
***SLC19A1***	rs1051266	GG	Reference		Reference		Reference	
		GA+AA	0.90 (0.21–3.78)	0.886	1.05 (0.35–3.17)	0.925	1.45 (0.57–3.69)	0.437
***SLCO1B1***	rs2306283	AA	Reference		Reference		Reference	
		AG+GG	1.33 (0.29–6.15)	0.712	1.12 (0.44–2.89)	0.809	1.75 (0.65–4.69)	0.265
	rs4149056	TT	Reference		Reference		Reference	
		TC+CC	0.72 (0.19–2.76)	0.633	0.65 (0.27–1.60)	0.348	0.83 (0.35–1.94)	0.664
	rs11045879	TT	Reference		Reference		Reference	
		TC+CC	0.83 (0.22–3.20)	0.790	0.63 (0.26–1.53)	0.306	0.86 (0.37–2.01)	0.724
***ABCB1***	rs2032582	GG	Reference		Reference		Reference	
		GT+GA+TT+AA	0.49 (0.11–2.25)	0.358	2.89 (0.62–13.44)	0.177	1.47 (0.54–4.01)	0.448
	rs1045642	CC	Reference		Reference		Reference	
		CT+TT	0.92 (0.15–5.78)	0.926	3.19 (0.40–25.62)	0.275	3.90 (0.50–30.26)	0.193
***ABCC2***	rs2804402	CC	Reference		Reference		Reference	
		CT+TT	1.83 (0.32–10.37)	0.493	0.57 (0.18–1.81)	0.338	0.99 (0.34–2.90)	0.987
	rs717620	GG	Reference		Reference		Reference	
		GA+AA	0.46 (0.10–2.07)	0.314	2.08 (0.75–5.79)	0.159	1.10 (0.46–2.64)	0.826
	rs2273697	GG	Reference		Reference		Reference	
		GA+AA	4.75 (1.15–19.65)	**0.031**	0.57 (0.22–1.49)	0.252	0.88 (0.37–2.08)	0.763
***ABCG2***	rs2231142	CC	Reference		Reference		Reference	
		CA+AA	2.29 (0.55–9.64)	0.258	2.13 (0.65–6.94)	0.209	1.57 (0.60–4.11)	0.361

a calculated using logistic regression;

b calculated by Cox proportional hazards model and adjusted for C-reactive protein level

**TABLE 3.** The influence of investigated polymorphisms on liver and gastrointestinal (GI) toxicity (N = 41)

**Gene**	**Polymorphism**		**Liver toxicity^a^**			**GI toxicity^a^**		

			**N (%)**	**OR (95 % CI)**	**p**	**N (%)**	**OR (95 % CI)**	**p**
***MTHFR***	rs1801133	CC	10 (45.5)	Reference		6 (27.3)	Reference	
		CT+TT	14 (73.7)	3.37 (0.90–12.6)	0.072	5 (26.3)	0.95 (0.24–3.81)	0.945
	rs1801131	AA	10 (58.8)	Reference		5 (29.4)	Reference	
		AC+CC	14 (58.3)	0.98 (0.28–3.46)	0.975	6 (25.0)	0.80 (0.20–3.22)	0.754
***MTHFD1***	rs2236225	GG	7 (46.7)	Reference		5 (33.3)	Reference	
		GA+AA	17 (65.4)	2.16 (0.59–7.90)	0.245	6 (23.1)	0.60 (0.15–2.46)	0.477
***TYMS***	rs34743033	2R/2R	7 (50.0)	Reference		2 (14.3)	Reference	
		2R/3R+3R/3R	17 (63.0)	1.7 (0.46–6.28)	0.426	9 (33.3)	3.00 (0.55–16.38)	0.205
***MTRR***	rs1801394	AA	6 (66.7)	Reference		1 (11.1)	Reference	
		AG+GG	18 (56.3)	0.64 (0.14–3.04)	0.577	10 (31.3)	3.64 (0.40–33.12)	0.252
***MTR***	rs1805087	AA	15 (60.0)	Reference		5 (20.0)	Reference	
		AG+GG	9 (56.3)	0.86 (0.24–3.06)	0.812	6 (37.5)	2.40 (0.59–9.82)	0.223
***SLC19A1***	rs1051266	GG	6 (50.0)	Reference		4 (33.3)	Reference	
		GA+AA	18 (62.1)	1.64 (0.42–6.36)	0.477	7 (24.1)	0.64 (0.15–2.77)	0.547
***SLCO1B1***	rs2306283	AA	9 (81.8)	Reference		3 (27.3)	Reference	
		AG+GG	15 (50.0)	0.22 (0.04–1.21)	0.081	8 (26.7)	0.97 (0.21–4.59)	0.696
	rs4149056	TT	17 (73.9)	Reference		4 (17.4)	Reference	
		TC+CC	7 (38.9)	0.23 (0.06–0.85)	**0.028**	7 (38.9)	3.02 (0.72–12.70)	0.131
	rs11045879	TT	18 (75.0)	Reference		5 (20.8)	Reference	
		TC+CC	6 (35.3)	0.18 (0.05–0.71)	**0.014**	6 (35.3)	2.07 (0.51–8.41)	0.308
***ABCB1***	rs2032582	GG	5 (55.6)	Reference		2 (22.2)	Reference	
		GT+GA+TT+AA	19 (59.4)	1.17 (0.26–5.20)	0.837	9 (28.1)	1.37 (0.24–7.88)	0.725
	rs1045642	CC	6 (66.7)	Reference		0 (0.0)	Reference	
		CT+TT	20 (57.1)	0.67 (0.11–4.13)	0.663	11 (31.4)	/	0.167^b^
***ABCC2***	rs2804402	CC	8 (88.9)	Reference		3 (33.3)	Reference	
		CT+TT	16 (50.0)	0.13 (0.01–1.12)	0.063	8 (25.0)	0.67 (0.14–3.30)	0.619
	rs717620	GG	15 (55.6)	Reference		3 (11.1)	Reference	
		GA+AA	9 (64.3)	1.44 (0.38–5.45)	0.591	8 (57.1)	10.67 (2.15–52.85)	**0.004**
	rs2273697	GG	17 (73.9)	Reference		6 (26.31)	Reference	
		GA+AA	7 (38.9)	0.23 (0.06–0.85)	**0.028**	5 (27.8)	1.09 (0.27–4.37)	0.903
***ABCG2***	rs2231142	CC	17 (56.7)	Reference		10 (33.3)	Reference	
		CA+AA	7 (63.6)	1.34 (0.32–5.56)	0.689	1 (9.1)	0.20 (0.02–1.79)	0.150

a calculated using logistic regression;

b calculated using Fisher’s exact test as there were no patients in one group

**TABLE 4.** The influence of *SLCO1B1* and *ABCC2* haplotypes on response rate, gastrointestinal (GI), and liver toxicity (N = 41)

**Gene**	**Haplotype**	**Estimated frequency**	**Response rate**		**GI toxicity**		**Liver toxicity**	

			OR (95 % CI)	*p*	OR (95 % CI)	*p*	OR (95 % CI)	*p*
***SLCO1B1***	ATT	0.47	Reference		Reference		Reference	
	GCC	0.22	0.98 (0.24–4.04)	0.973	2.63 (0.61–11.37)	0.195	0.17 (0.03–0.98)	**0.048**
	GTT	0.29	1.94 (0.66–5.74)	0.229	0.37 (0.07–1.93)	0.237	0.46 (0.13–1.63)	0.230
***ABCC2***	CGG	0.28	Reference		Reference		Reference	
	CAG	0.20	0.30 (0.05–1.71)	0.175	5.67 (1.64–19.66)	**0.006**	1.06 (0.21–5.05)	0.941
	TGG	0.28	0.29 (0.06–1.46)	0.133	0.33 (0.07–1.61)	0.171	0.74 (0.18–3.07)	0.680
	TGA	0.24	1.59 (0.39–6.43)	0.519	0.88 (0.23–3.39)	0.847	0.29 (0.06–1.27)	0.099
