# Supplementary material for: Polymorphisms in folate pathway and pemetrexed treatment outcome in patients with malignant pleural mesothelioma
Source: Radiol Oncol. 2014 Apr 25;48(2):163–72. doi: 10.2478/raon-2013-0086 (PMC4078035; doi:10.2478/raon-2013-0086)
Supplement: Supplementary file 1 [file raon-2014-issue2-0162_supp1.pdf]

SUPPLEMENTAL TABLE 1. Distribution of genotype frequencies in patients with malignant pleural mesothelioma (N = 41)

Gene	Polymorphism	Genotype	N (%)
MTHFR	**rs1801133**	CC	22 (53.7)
	C677T	CT	15 (36.6)
	Ala222Val	TT	4 (9.8)
	**rs1801131**	AA	17 (41.5)
	A1298C	AC	18 (43.9)
	Glu429Ala	CC	6 (14.6)
MTHFD1	**rs2236225**	GG	15 (36.6)
	G1958A	GA	19 (46.3)
	Arg653Gln	AA	7 (17.1)
TYMS	**rs34743033**	2R/2R	14 (34.1)
	5' UTR	2R/3R	16 (39.0)
	2R>3R	3R/3R	11 (26.8)
MTRR	**rs1801394**	AA	9 (22.0)
	A66G	AG	18 (43.9)
	Ile22Met	GG	14 (34.1)
MTR	**rs1805087**	AA	25 (61.0)
	A2756G	AG	15 (36.6)
	Asp919Gly	GG	1 (2.4)
SLC19A1	**rs1051266**	GG	12 (29.3)
	G80A	GA	22 (53.7)
	Arg27Cys	AA	7 (17.1)
SLCO1B1	**rs2306283**	AA	11 (26.8)
	A388G	AG	18 (43.9)
	Asn130Asp	GG	12 (29.3)
	**rs4149056**	TT	23 (56.1)
	T521C	TC	16 (39.0)
	Val174Ala	CC	2 (4.9)
	**rs2900478**	TT	24 (58.5)
	intron	TA	15 (36.6)
	T>A	AA	2 (4.9)
	**rs11045879**	TT	24 (58.5)
	intron	TC	15 (36.6)
	T>C	CC	2 (4.9)
ABCB1	**rs2032582**	GG	9 (22.0)
	G2677T/A	GT+GA	20 (48.8)
	Ala893Ser/Thr	TT+AA	12 (29.3)
	**rs1045642**	CC	6 (14.6)
	C3435T	CT	21 (51.2)
	Ile1145Ile	TT	14 (34.1)
ABCC2	**rs2804402**	CC	9 (22.0)
	5' UTR	CT	21 (51.2)
	-1019A>G	TT	11 (26.8)
	**rs717620**	GG	27 (65.9)
	5' UTR	GA	12 (29.3)
	-24C>T	AA	2 (4.9)
	**rs2273697**	GG	23 (56.1)
	G1249A	GA	16 (39.0)
	Val417Ile	AA	2 (4.9)
ABCG2	**rs2231137**	GG	40 (97.6)
	G34A	GA	1 (2.4)
	Val12Met	AA	/
	**rs2231142**	CC	30 (73.2)
	C421A	CA	11 (26.8)
	Gln141Lys	AA	/
ABCC4	**rs2274407**	CC	37 (90.2)
	C912A	CA	4 (9.8)
	Lys304Asn	AA	/
