# Supplementary material for: Polymorphisms in folate pathway and pemetrexed treatment outcome in patients with malignant pleural mesothelioma
Source: Radiol Oncol. 2014 Apr 25;48(2):163–72. doi: 10.2478/raon-2013-0086 (PMC4078035; doi:10.2478/raon-2013-0086)
Supplement: Supplementary file 2 [file raon-2014-issue2-0162_supp2.pdf]

SUPPLEMENTAL TABLE 2. The influence of selected polymorphisms on overall toxicity, hematological and renal toxicity

Gene	Polymorphism	Genotype	Overall toxicity			Hematological toxicity			Renal toxicity		
			N (%)	OR (95 % CI) [a]	p^a	N (%)	OR (95 % CI) [a]	p^a	N (%)	OR (95 % CI) [a]	p^a
MTHFR	rs1801133	CC	17 (77.3)	Reference		10 (45.5)	Reference		11 (50.0)	Reference	
		CT+TT	17 (89.5)	2.50 (0.43-14.71)	0.311	9 (47.4)	1.08 (0.32-3.70)	0.902	7 (36.8)	0.58 (0.17-2.04)	0.399
	rs1801131	AA	15 (88.2)	Reference		10 (58.8)	Reference		6 (35.3)	Reference	
		AC+CC	19 (79.2)	0.51 (0.09-2.99)	0.453	9 (37.5)	0.42 (0.12-1.50)	0.181	12 (50.0)	1.83 (0.55-6.57)	0.352
MTHFD1	rs2236225	GG	14 (93.3)	Reference		9 (60.0)	Reference		8 (53.3)	Reference	
		GA+AA	20 (76.9)	0.24 (0.03-2.20)	0.206	10 (38.5)	0.42 (0.11-1.53)	0.187	10 (38.5)	0.55 (0.15-1.98)	0.358
TYMS	rs34743033	2R/2R	11 (78.6)	Reference		5 (35.7)	Reference		7 (50.0)	Reference	
		2R/3R+3R/3R	23 (85.2)	1.57 (0.30-8.25)	0.595	14 (46.3)	1.94 (0.51-7.32)	0.329	11 (40.7)	0.69 (0.19-2.52)	0.572
MTRR	rs1801394	AA	6 (66.7)	Reference		3 (33.3)	Reference		2 (22.2)	Reference	
		AG+GG	28 (87.5)	3.5 (0.62-19.50)	0.158	16 (50.0)	2.00 (0.43-9.42)	0.381	16 (50.0)	3.5 (0.63-19.50)	0.153
MTR	rs1805087	AA	19 (76.0)	Reference		11 (44.0)	Reference		9 (36.0)	Reference	
		AG+GG	15 (93.8)	4.74 (0.51-47.73)	0.170	8 (50.0)	1.27 (0.36-4.48)	0.707	9 (56.3)	2.29 (0.63-8.23)	0.206
SLC19A1	rs1051266	GG	9 (75.0)	Reference		4 (33.3)	Reference		3 (25.0)	Reference	
		GA+AA	25 (86.2)	2.08 (0.39-11.18)	0.392	15 (51.7)	2.14 (0.53-8.72)	0.287	15 (51.7)	3.2 (0.72-14.35)	0.126
SLCO1B1	rs2306283	AA	9 (81.8)	Reference		5 (45.5)	Reference		4 (36.4)	Reference	
		AG+GG	25 (83.3)	1.11 (0.18-6.78)	0.909	14 (46.7)	1.05 (0.26-4.20)	0.945	14 (46.7)	1.53 (0.37-6.35)	0.557
	rs4149056	TT	20 (87.0)	Reference		13 (56.5)	Reference		10 (43.5)	Reference	
		TC+CC	14 (77.8)	0.53 (0.10-2.72)	0.443	6 (33.3)	0.39 (0.11-1.38)	0.144	8 (44.4)	1.04 (0.30-3.60)	0.951
	rs11045879	TT	21 (87.5)	Reference		13 (54.2)	Reference		10 (41.7)	Reference	
		TC+CC	13 (76.5)	0.46 (0.09-2.42)	0.362	6 (35.3)	0.46 (0,13-1.66)	0.236	8 (47.1)	1.24 (0.36-4.35)	0.732
ABCB1	rs2032582	GG	7 (77.8)	Reference		5 (55.6)	Reference		3 (33.3)	Reference	
		GT+GA+TT+AA	27 (84.4)	1.54 (0.25-9.70)	0.601	14 (43.8)	0.62 (0.14-2.76)	0.532	15 (46.9)	1.77 (0.38-8.32)	0.473
	rs1045642	CC	5 (83.3)	Reference		3 (50.0)	Reference		4 (66.7)	Reference	
		CT+TT	29 (82.9)	0.97 (0.10-9.84)	0.977	16 (45.7)	0.84 (0.15-4.76)	0.846	14 (40.0)	0.33 (0.05-2.07)	0.239
ABCC2	rs2804402	CC	9 (100.0)	Reference		2 (22.2)	Reference		2 (22.2)	Reference	
		CT+TT	25 (78.1)	/	0.315[b]	17 (53.1)	3.97 (0.71-22.11)	0.116	16 (50.0)	3.50 (0.63-19.50)	0.153
	rs717620	GG	21 (77.8)	Reference		14 (51.9)	Reference		13 (48.1)	Reference	
		GA+AA	13 (92.9)	3.71 (0.40-34.44)	0.248	5 (35.7)	0.52 (0.14-1.95)	0.329	5 (35.7)	0.60 (0.16-2.26)	0.449
	rs2273697	GG	22 (95.7)	Reference		11 (47.8)	Reference		9 (39.1)	Reference	
		GA+AA	12 (66.7)	0.09 (0.01-0.85)	**0.035**	8 (44.4)	0.87 (0.25-3.01)	0.829	9 (50.0)	1.56 (0.45-5.41)	0.487
ABCG2	rs2231142	CC	26 (86.7)	Reference		15 (50.0)	Reference		13 (43.3)	Reference	
		CA+AA	8 (72.7)	0.41 (0.08-2.23)	0.303	4 (36.4)	0.57 (0.14-2.37)	0.440	5 (45.5)	1.09 (0.27-4.37)	0.903

[a]calculated using logistic regression; [b]calculated using Fisher's exact test as there were no patients in one group; CI = confidence interval; OR = odds ratio.
